# Intraspecific Variability of Wild-Growing Common Valerian (*Valeriana officinalis* L.)

**DOI:** 10.3390/plants11243455

**Published:** 2022-12-09

**Authors:** Katarzyna Barbara Bączek, Olga Kosakowska, Maja Boczkowska, Paulina Bolc, Rafał Chmielecki, Ewelina Pióro-Jabrucka, Kavana Raj, Zenon Węglarz

**Affiliations:** 1 Department of Vegetable and Medicinal Plants, Institute of Horticultural Sciences, Warsaw University of Life Sciences SGGW, 159 Nowoursynowska Street, 02-776 Warsaw, Poland; 2 Plant Breeding and Acclimatization Institute, National Research Institute, 05-870 Radzików, Poland; 3 Center for Biological Diversity Conservation in Powsin, Polish Academy of Sciences Botanical Garden, 02-973 Warszawa, Poland; 4 Martin Bauer Polska sp. z o.o., 63-230 Witaszyce, Poland

**Keywords:** *Valeriana officinalis*, wild-growing populations, landrace, intraspecific variability, underground and aboveground organs, chemical diversity, molecular variability, gene pools, NGS-DArT-seq method

## Abstract

Common valerian (*Valeriana officinalis* L.) is an important medicinal plant revealing sedative, hypotensive, anti-spasmodic and anxiolytic activity. The purpose of the study was to determine the intraspecific variability of the common valerian growing wild in Poland and the ‘Lubelski’ landrace, as to their developmental traits, chemical composition and selected genetic parameters. Both wild-growing populations (19) and the landrace (1) were evaluated under ex situ conditions. Observations of the underground organs parameters, both developmental and chemical (according to the European Pharmacopoeia) were carried out in the first year of the plant’s development, while the characteristics of the aboveground organs, followed by the sowing value of seeds (according to the International Seed Testing Association)—in the second year. The genetic analyses were performed using the NGS-DArT-seq method. Results indicate the presence of five different gene pools covering the regions of population’s origin, with a gene flow within and between them. A high level of developmental and chemical variabilities among the wild-growing populations was noticed, however without a clear relation to the region of the origin. The mass of underground organs ranged from 107.4 to 403.6 g FW × plant^−1^ with the content of sesquiterpenic acids at the level of 0.004–0.094%. Population no 18 was distinguished by the highest content of sesquiterpenic acids and the relatively high mass of underground organs, followed by the admixture of the gene pool, typical for the ‘Lubelski’ landrace. Unlike the ‘Lubelski’ landrace, the wild-growing populations were characterized by a high amount of an essential oils (3.90 to 10.04 mL/kg), which may be promising from the perspective of their potential use. In turn, the sowing value of the seeds obtained from the populations, expressed as the germinability, was rather low (25.25–62.25%).

## 1. Introduction

The common valerian (*Valeriana officinalis* L.) (Caprifoliaceae family) is a perennial plant, native to Europe and Asia. It grows mainly on wet meadows, water banks, low peat bogs and in moist forests [1]. The species is recognized as highly polymorphic, in respect of the genome size, developmental traits and chemical composition. Many subspecies, types and hybrids followed by four ploidy levels (di-, tetra-, hexa and octoploidy) have been noticed within the species. Taking this into account, the common valerian is regarded as a collective taxon [2,3,4]. Its underground organs (*Valerianae radix*)—rhizomes with roots and stolons—are listed in the European Pharmacopoeia and classified by the European Medicines Agency as a traditional herbal drug [5,6]. This raw material contains an essential oil with typical mono- and sesquiterpenes (mainly borneol, camphene, myrtenol, β-caryophyllene and its derivatives); valerian cyclopentanoid sesquiterpenes, such as valerenal, valeranone, valerenol and the less volatile sesquiterpene acids, valerenic, acetoxyvalerenic and hydroxyvalerenic acids [7,8,9]. According to the EP requirements, dried, whole or fragmented roots should contain no less than 0.17% of sesquiterpene acids expressed as valerenic acid and 4 mg/kg of the essential oil. Other biologically active compounds present in this raw material are esterified iridoid-monoterpenes so-called valepotriates (i.e., valtrate and isovaltrate) [5,6]. Valerian root shows sedative, hypotensive, anti-spasmodic and anxiolytic activities, and has been used in traditional medicine as a sleep promoting agent in the treatment of nervous states and anxiety. The anxiolytic activity is probably associated with the synergistic action of valerenic acid and valepotriates leading to the barbiturate-like effect through GABA and the serotonergic systems modulation [10,11,12,13]. Recently, valerian root has been considered as a milder alternative or a possible substitute for stronger synthetic sedatives, such as benzodiazepines. Considering the recent worldwide increase in stress-derived lifestyle diseases, including depression, followed by the benzodiazepine drug addictions, the need for natural remedies has grown significantly. Thus, valerian root appears to be one of the bestselling herbal raw materials in Europe and the USA today [14,15,16]. 

In the past, valerian roots were collected from natural sites. However, due to the growing demand for this raw material, as well as the high requirements of the herbal industry, common valerian has been introduced into cultivation on the commercial scale in northern parts of Europe and America. So far, a few landraces have been available for stakeholders in Poland: ‘Polka’, ‘Norweski’ and ‘Lubelski’ [17,18]. It is worth noting that the cultivating of *V.officinalis*, as well as other medicinal and aromatic plants, is at its initial stages, when compared to other crops [19,20]. Regarding a polymorphic character of the common valerian, wild-growing populations of this species may be a huge source of genes necessary in future cultivating programmes. Up to now, data on the intraspecific variabilities of the common valerian growing wild in Europe, are rather scarce and focus mainly on the ploidy level [3,21,22,23]. 

It seems that the climate changes observed in recent years, followed by the rapid agricultural and urban development, as well as the uncontrolled logging of forests, constitute serious threats for the natural habitats of plants, resulting in the progressive loss of populations. According to Kostarkiewicz-Gierałt [1], the common valerian belongs to taxa growing wild in moist habitats which suffer from degradation caused mainly by improper water management. Thus, it is crucial to monitor the natural recourses of such species, both on the environmental, molecular and chemical levels. In recent decades, a variety of molecular techniques and genetic markers have been extensively developed to estimate the genetic diversity of plants. The molecular markers, such as AFLP, RAPD, SAMPL and SSR, have been applied to detect the DNA polymorphism between/within species [3,20,24,25,26]. However, these techniques were not selective enough. Recently, the next-generation sequencing technology (including the DArTseq method) has appeared as a modern and effective tool for plant genotyping. This ultra-high-throughput, scalable and high-speed technology can be applied to organisms with an unknown genome sequence. Until now, DArTseq has been successfully applied to several plant species, in particular crops [27]. Only a few studies have been published on the use of this product in the genotyping of wild and medicinal plant species. In our previous work, we investigated the usefulness of the DArT-seq technique for the genotyping of *V. officinalis* [28]. The aim of the present work was to determine the intraspecific variability of the common valerian growing wild in Poland, in terms of the developmental traits, chemical composition (essential oils and sesquiterpene acids’ content) and selected genetic parameters obtained by the DArT-seq application.

## 2. Results and Discussion

### 2.1. Developmental and Chemical Variabilities

It was shown that the examined populations differed both in the developmental and chemical traits. Regarding the first year of the plants’ development, it was observed that the diameter of the rhizome was at a level of 1.7–6.0 cm (CV = 0.24). The mass of underground organs ranged from 107.4 to 403.6 g FW × plant^−1^, CV = 0.35 (Table 1). Populations No. 5 and 18 (Bieszczady Mountains and Mazovian Lowland, respectively) were distinguished by the highest mass of underground organs (in total 403.6 and 331.2 g FW × plant^−1^), comparable to the ‘Lubelski’ landrace (375.0 g FW × plant^−1^), which is considered a high-yielding form [17]. 

The investigated raw materials were subjected to a chemical analysis, to determine the total content of sesquiterpenic acids (expressed as valerenic acid) and essential oils, which are listed in the EP monograph as quality markers of Valeriana radix [6]. It was shown that the average content of the sesquiterpenic acids in the underground organs of the populations examined, was visibly lower (average 0.014%), in comparison to the ‘Lubelski’ landrace (0.175%) and did not meet the pharmacopeial threshold (no less than 0.17%). Nevertheless, the content of these compounds was extremely variable (0.004–0.094%) and significantly differentiated the populations (CV = 1.35) (Table 1). Their highest amount was noticed in populations No. 18 (0.094%) and 5 (0.031%). It shall be underlined that these populations were also distinguished by the highest mass of underground organs. The results correspond to those obtained by other authors. Studies by Nakurte et al. [16] show that the content of sesquiterpenic acids in the wild-growing common valerian from Latvia, ranged from 0.002 to 0.014%. In turn, this value for the cultivated forms (including ‘Lubelski’) varied from 0.119 to 0.350%. Authors agreed that the content of sesquiterpenic acids in the underground organs of the common valerian is variable and depends both on the genetic and ontogenetic factors [16,18,29,30]. 

In our work, the content of the essential oils in the underground organs of the populations investigated, varied between 3.9 and 10.4 mL/kg (CV = 0.27), while in the case of the ‘Lubelski’ landrace, it was slightly lower (6.3 mL/kg) (Table 1). A similar relationship was observed earlier by Narkute at al. [16], where the range of 8.5–11.3 mg/kg was given in the case of the wild-growing forms, and 6.8–8.7 mg/kg in the cultivated ones. Raal et al. [7] demonstrated that the roots of the valerian grown in Estonia contain from 0.28 to 1.16% of essential oils. 

In the second year of the plant’s development, the populations investigated were characterized with regards to the traits of the aboveground organs. The height of the plants ranged from 110 (population No. 2) to 143 cm (population No. 18) (CV = 0.07), while the number of flowering shoots per plant was from five (population No. 2 and 16) to 15 (population No. 1) (CV = 0.27). These features differentiated the populations at a relatively low degree, and corresponded well with the ‘Lubelski’ landrace (128 cm; 13, respectively) (Table 2). According to the literature data, the species reaches the height of up to 200 cm [1,20]. Morteza et al. [31] showed that the number of shoots per plant ranged from about five to eight. In our study, the lushness of the plant was described as high, in the case of population No. 16; in population 18 and 19, it was characterized as medium; and in population No. 1, it was described as low. Other populations appeared to be heterogenic and represented mixed types (Table 2). The colour of shoots, as well as the flower buds and petals were also differentiated among the populations examined. Here, the anthocyanin colouring of the shoots was not found in populations No. 4 and 5; it was of a medium intensity in populations No. 11 and 15; and variable (absent/medium intensity/intense) in the others. Population no 19 was distinguished by white flower buds, populations No. 2, 3, 8, 9, 10 had pale pink buds, and population No. 1 had pink buds. The colour of the petals was white (populations No. 12, 13, 18, 19), pale pink (populations No. 2 and 10) and pink (population No. 1). As regards to the traits of the leaves, populations No. 2, 9, 14, 15 and 17 were characterized by medium-deep teething of the leaf margin, while the others were intermediate, both shallow, medium deep and deep. In the majority of the populations, the width of the leaf section was narrow, and only in the case of population No. 4, was it medium-wide. This feature, in other populations, was described as narrow and medium-wide. It was observed that the ‘Lubelski’ landrace did not differ visibly from the populations investigated, as regards to the above-mentioned traits. The landrace was characterized by a medium and low lushness, white and pale pink flower buds and petals, shallow and medium-deep teething of the leaf margin and a narrow width of the leaf section. Its shoots were variable, with regards to the anthocyanin colouring level (Table 2). 

In Central Europe, the common valerian blooms from June to July, when its hermaphrodite flowers are frequently visited by pollinators, especially hoverflies of the Eristalis genus [32]. The species is allogamous (cross-pollinated), with a dichogamy as a mechanism protecting single flowers (but not the whole inflorescences) against self-pollination. The seeds are dark brown, approximately 3 mm long, with longitudinal ribs on the surface [33,34]. At the time of the seed ripening, the seeds bear a pappus; the lanceolate-oblong achenes are wind-dispersed. As regards to this trait, in the agricultural practice, the common valerian is usually propagated generatively, thus in the present work, we investigated the parameters of the seeds that are associated with their sowing value. It was observed that the mass of one thousand seeds ranged from 0.1380 g (population No. 5) to 0.4785 g (population No. 2) (Table 3). Bomme et al. [33] claim that this parameter oscillates from 0.4 to 1.1 g. In our study, the germinability varied from 25.25% (population No. 14) to 62.25% (population No. 16) (Table 3). Such a low and uneven germination is typical for wild-growing plants, since this phenomenon is one of their adaptation/survival strategies [1,35,36,37]. When given the germination rate, population No. 15 was distinguished as the fastest (4.64 days), while population No. 6 had the slowest (13 days) germination. It is worth noting that the ‘Lubelski’ landrace was characterized by both a higher mass of one thousand seeds (0.6463 g) and a higher germination ability (81.25%), in comparison with the wild-growing populations (Table 3).

### 2.2. Genetic Variability

The results described above indicate a remarkable polymorphism, both morphologically and chemically, within the common valerian species. However, no clear relationship between the traits examined and the geographical location of the populations was recognized. The phenotypic plasticity observed may be associated with the ploidy level of the common valerian, corresponding with the ‘basic types’ described within the species [3,4,23,38,39,40]. The ploidy level is regarded as an important determinant of the various developmental and chemical traits of plants [41,42,43]. The common valerian that grows wild in Europe is predominantly represented by a diploid form, however, polyploids (tetra-, hexa-, octoploids) sporadically occur at natural sites, as well. The results of our previous study showed that the populations examined are diploids, contrary to the tetraploid ‘Lubelski’ landrace [28]. According to Noller [44], tetraploids are able to produce a higher biomass, in comparison to diploids. This is visibly seen in the case of the ‘Lubelski’ landrace, that is characterized by wide leaves and a large number of thick roots with an appropriate diameter and weight. Taking into account such traits, which are important from a practice viewpoint, in recent years, the natural resources of the common valerian have been screened mainly towards karyotype forms [3,21,23]. However, the huge variability has also been noticed irrespective of the ploidy level, e.g., diploids occurring in Europe can create at least two botanical varieties: Valeriana officinalis var. tenuifolia Vahl. and Valeriana officinalis var. latifolia Vahl. [45]. Up till now, the molecular basics of this variability have not been recognized yet. In the present work, we have applied advanced molecular techniques, based on next generation sequencing (NGS), to monitor the genetic parameters of the investigated common valerian populations, including their gene pool and the gene flow between them.

**Figure 1:**
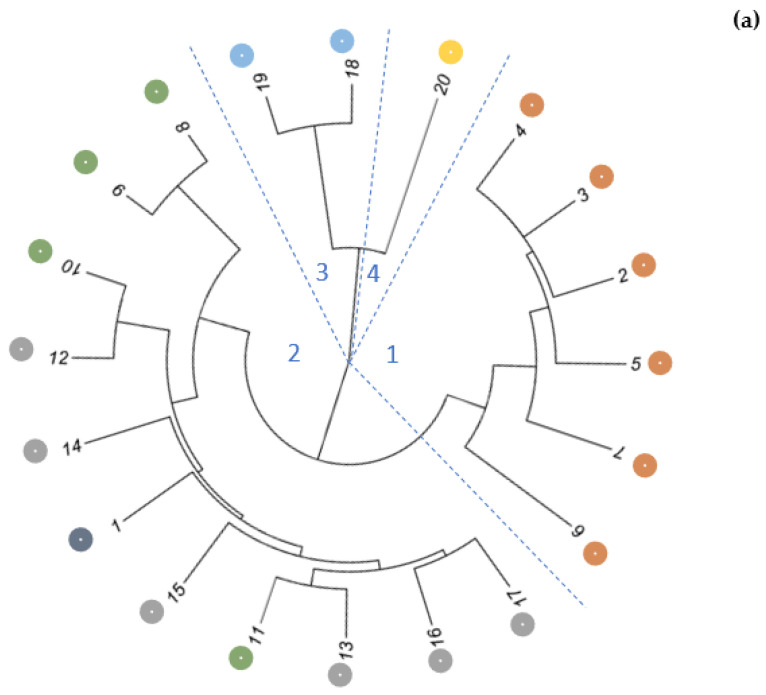

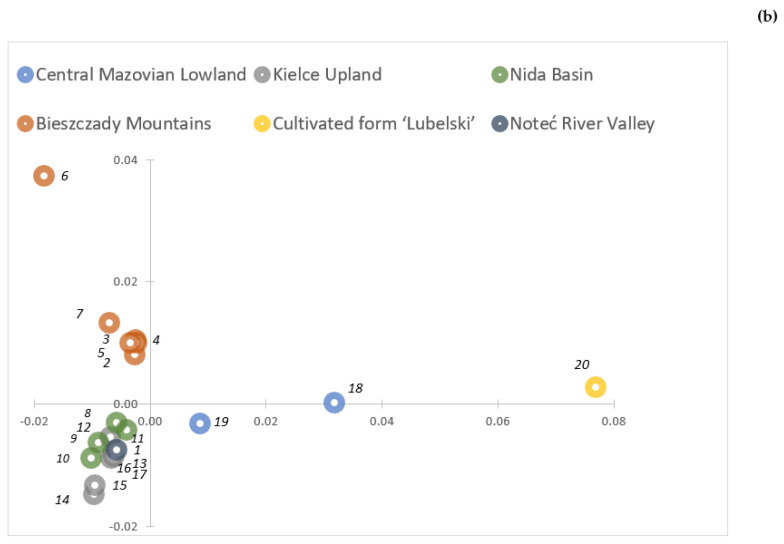
Genetic differentiation of 20 Valeriana officinalis accessions. (**a**) Results of the agglomerative hierarchical clustering, based on the unbiased Nei coefficient; (**b**) Results of the principal coordinate analysis for the first two PCs. The region of origin was marked with colors.

A genetic analysis was performed on the basic 23507 SNP loci, meeting the following criteria, i.e., a reproducibility above 90% and a call rate above 95%. The average value of the PIC was 0.181. The genetic distance of the studied accessions ranged from 0.019 (population No. 10–population No. 12) to 0.104 (the ‘Lubelski’ landrace–population No. 6). The Mantel test showed that the genetic distance was moderately positively correlated with the geographical distance (r = 0.304, *p* <0.0001) and weakly correlated with the annual precipitation (r = 0.280, *p* <0.0001). There was no significant correlation between the genetic distance and the average annual temperature and the altitude of the collection sites. The agglomerative hierarchical clustering (AHC), based on the Ward method, indicated the presence of four main clusters composed of six, eleven, two and one accessions, respectively (Figure 1a). The first group included only wild populations that originated from the Bieszczady Mountains. The second, the largest group, included populations from three regions, i.e., the Nida Basin, the Kielce Upland and the Noteć Valley. The third cluster contained populations from the Central Mazovian Lowland. The cultivated form ‘Lubelski’ was separated from the wild-growing populations. 

The principal coordinate analysis (PCoA), based on the genetic distance, revealed that the first three PCs together accounted for 58.8% of the total variation. The first, second and third PCs accounted for 38.6%, 13.5% and 6.7%, respectively. A two-dimensional PC plot (PC1 vs. PC2) of the common valerian accessions is shown in Figure 1b. It revealed a similar grouping pattern to the AHC plot. PC1 separated the ‘Lubelski’ landrace from the wild-growing populations and also indicated the distinctiveness of the population from the Central Mazovian Lowland, from the others. PC2 separated two groups from the remaining wild populations. The first one contained only the populations from the Bieszczady Mountains. However, a considerable separation of population No. 6 was observed. The second group consisted of 11 populations, and it corresponded exactly to the second group displayed on the dendrogram.

**Figure 2:**
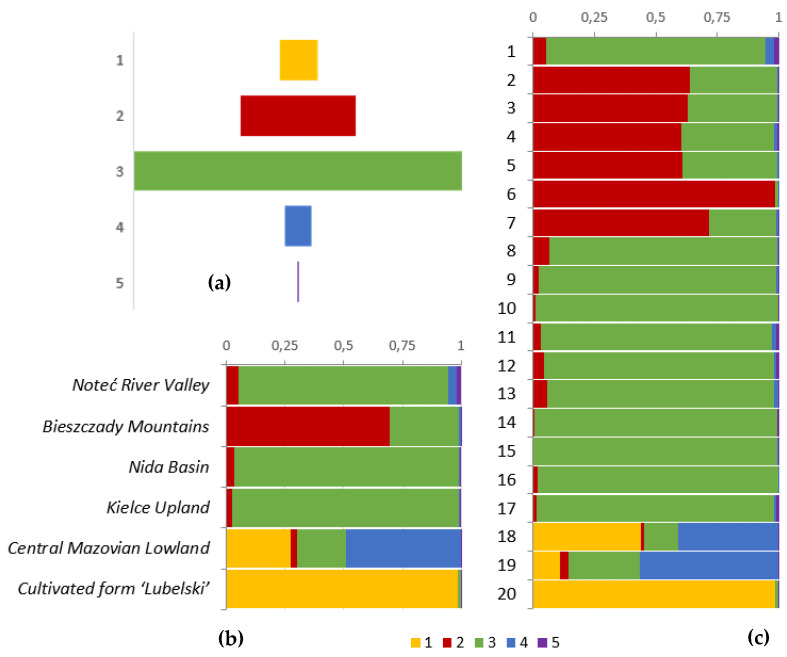
Results of the genetic clustering of 20 accessions of *V. officinalis*, based on the DArTseq derived SNPs. (**a**) Content of the individual gene pools in the investigated populations of *V. officinalis*.; (**b**) Bayesian inference of the population structure at K = 5, using STRUCTURE for the 20 populations. (**c**) Bayesian inference of the population structure at K = 5, using STRUCTURE for the geographic regions.

A structural analysis of the populations using the Bayesian model was carried out using the STRUCTURE software. For each accession, the proportion of its genome derived from the different clusters was estimated and the accessions were assigned to a cluster when 70% or more of their inferred genome belonged to the cluster, whereas the accessions with a lower percentage were considered to be admixed. The second-order likelihood delta K calculated, following Evanno et al. [46], indicated that within 20 accessions under study, five distinct gene pools were present. The clusters contributed in 7.6%, 22.7%, 63.6%, 5.6% and 0.5% of the total genetic makeup of the common valerian, respectively (Figure 2a). The cultivated form ‘Lubelski’ was qualified as pure and representing the first gene pool (Figure 2b). This pool was also found in populations from the Central Mazovian Lowland and constituted 27.2% of their genetic makeup. The second gene pool occurred mainly in the Bieszczady region and constituted 69.7% of the genotype of the indigenous populations. In other geographical regions, its contribution was up to 5%. The third detected pool, which had the biggest contribution to the sample examined, occurred mainly in three regions, i.e., the Noteć River Valley, the Nida Basin and the Kielce Upland, and its participation in the genetic makeup there was about 90%. In the two other regions, it was at a much lower level, i.e., about 25%. The fourth gene pool was present mainly in populations from the Central Mazovian Lowlands and determined about 50% of their genotype. In other regions, its contribution in the genotype was negligible. The fifth gene pool had a very small impact on all studied accessions. Fourteen out of 20 investigated accessions were considered as pure. Four populations from the Bieszczady Mountains and two populations from the Central Mazovian Lowland have been recognized as admixed (Figure 2c).

The structure analysis of the investigated wild-growing populations revealed the presence of different gene pools in the studied geographical regions. The most distinctive were populations No. 2, 3, 4, 5, 6 and 7, inhabiting the Bieszczady Mountains. This is most likely due to the presence of natural barriers in the topography. However, they do not provide full isolation and there is gene flow both within and between the geographical regions. Moreover, on the basis of the obtained picture of the population structure, which indicates a high genetic similarity of the populations from the Noteć River Valley, the Nida Basin and the Kielce Upland, it can be concluded that the common valerian occurs in the form of a metapopulation with free gene flow in a significant area of Poland. Such an assumption is supported by the widespread occurrence of the common valerian throughout the entire country, but its verification will be possible only after examining a larger number of wild-growing populations representing all geographical regions of Poland. Interestingly, there is a genetic distinctness of the ‘Lubelski’ landrace, from the wild-growing populations. The background of this is probably a different ploidy level, as it was mentioned before. In the wild-growing populations from the Middle Mazovian Lowland, a significant admixture of the gene pool, typical for the cultivated form, was observed. It can therefore be assumed that we are dealing with the uncontrolled spread of the cultivated form beyond the plantation area. Native species are not being surveyed in this context. However, the ploidy analysis did not reveal the presence of tetraploid individuals in these two populations. Therefore, we are probably faced with hybrids between the diploid and tetraploid forms, which have stabilized at the level of 2n = 14. On the basis of the results obtained, it is impossible to say whether the hybridization took place in the Central Mazovian Lowland as a result of crossing the cultivated form with the wild plants or whether there was a hybridization of the two naturally occurring forms in the Lublin region, from where the cultivated form was selected. The hybrids among the forms with different ploidy levels have been proven to exist within *V. officinalis*. The admixture of 55% was described in populations with di- and tetraploid specimens [3,23,40,47,48].

Due to the presence of the gene flow between populations, the value of the correlation between the genetic and geographical distances was only at a moderate level. There is a report in which it is demonstrated that there is a significant link between the geographical distance and the relative size of the genome [3]. It seems important to also observe a correlation between the genetic distance and the difference in the annual precipitation levels in the collection sites. *V. officinalis* is a species related to fresh habitats and margins of water courses and, as can be seen, water availability is, in addition to the spatial isolation, a factor determining the genetic makeup of the species in the region. Disturbance in the water conditions of the habitat, as a result of climatic changes, may lead to a significant reduction of the occurrence of this species in Poland.

## 3. Materials and Methods

In agricultural practice, the underground organs of the common valerian are usually harvested in the first autumn of the plant’s vegetation. During this period, the plants are at the vegetative stage and form a basal rosette of pinnatisect leaves with rhizomes and numerous roots. In the following year, the plants form flowering shoots and seeds. In our work, observations were carried out in the first and second years of the plant’s development, covering the terms of both the raw and reproductive materials.

### 3.1. Plant Material

**Figure 3:**
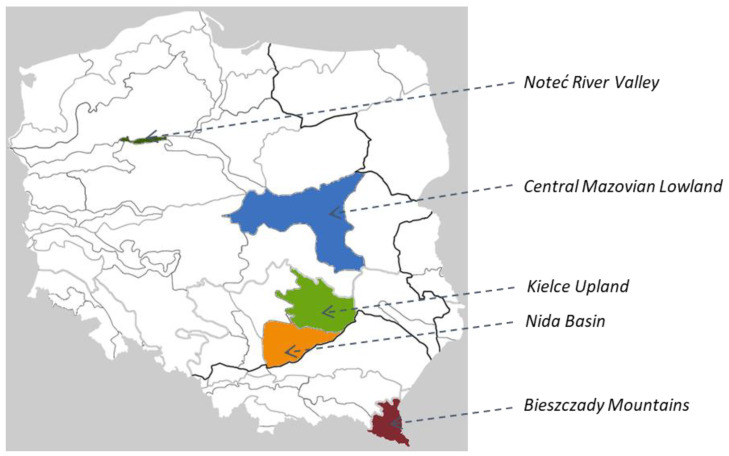
Geographical localization of the natural sites of the common valerian populations.

The objects of the study were 19 wild-growing populations and ‘Lubelski’ landrace (20 accessions) of the common valerian introduced to ex situ conditions. The seeds of wild-growing populations were collected in 2015 from natural sites covering five geographical regions of Poland, i.e., the Noteć River Valley, the Central Mazovian Lowland, the Kielce Upland, the Nida Basin and the Bieszczady Mountains (Table 4, Figure 3). The locations of the collection sites were within the range of N 49 21 325–N 53 03 120 latitude and E 17 11 506–E 22 15 037 longitude. The altitude was within the range of 50–600 m above the mean sea level (MASL). The annual precipitation in the places of origin ranged from 521 mm to 874 mm and the average annual temperature ranged from 6.36 to 8.25 °C.

In February 2017, the seeds of the wild-growing populations and the ‘Lubelski’ landrace were sown (by a single seed technique) into multi-pots filled with a peat substrate, in a greenhouse. In May, the well rooted seedlings (30 per accession) were planted in 60 × 40 cm spacing in a field located at the Experimental Station of the Vegetable and Medicinal Plant Department, WULS-SGGW (N 52 10 180; E 21 05 23).

The seed specimens were deposited in the National Centre for Plant Genetic Resources (Polish Gene Bank), under the accession numbers given in Table 4.

### 3.2. Developmental Characteristics

In the first year of the plants’ development, at the beginning of plants’ dormancy (October 2017), the underground organs were collected from 10 randomly chosen plants, per accession. The following observations and measurements were carried out: the diameter of rhizomes (cm) and mass of the roots and rhizomes (g FW per plant). The raw materials were purified, dried at 35 °C, ground and subjected to the chemical analysis (Section 3.3.). 

In the second year of the plants’ development, when they were in full bloom (June 2018), observations on the parameters of the aboveground organs were determined, namely: the plant height (cm), the number of flowering shoots per plant, plant lushness, the anthocyanin colouring of the shoots, the colour of the petals, the teething of the leaf margin and the width of the leaf sections. These traits were assessed using a 3-step-scale, which was previously elaborated. At the seed ripening stage (August 2018), the seeds were collected, purified and evaluated as to their sowing value (Section 3.4). The observations were conducted on 10 randomly selected plants.

### 3.3. Chemical Analysis 

The analyses (essential oil and sesquiterpenes acid content) were carried out, according to the European Pharmacopoeia, *Valerianae radix* monograph 07/2015:0453 [6]. The analyses were performed in triplicate. In order to obtain the essential oils, 40 g of air-dried powdered raw material was used for hydrodistillation, for 4 h, using a Clevenger-type apparatus. Due to the determination of the sesquiterpenic acid content, the methanolic extract was prepared (1.5 g of air-dried powdered raw material per 50 mL of methanol). The obtained extract was separated by HPLC Shimadzu Prominence chromatograph, equipped with autosampler SIL–20AC HT, photodiode array detector SPD–M20A and LCsolution 1.21 SP1 chromatography software (Shimadzu, Kyoto, Japan).

### 3.4. Seed Evaluation

The following analyses were carried out according to the International Seed Testing Association (ISTA) protocols [49]: 1000 seeds mass (g), germinability (%) and germination rate (days).

#### 3.4.1. Seed Mass Test

The test was determined by the calculation of an average of eight replications containing 100 seeds, multiplied by 10.

#### 3.4.2. Germinability and Germination Rate Tests

The tests were determined in climatic chambers (MLR 350, Sanyo), in the controlled conditions: PPFD = 150 µE/m^2^×s; temp. 21 °C for 24 h. Four replications were used with 100 seeds each. The seeds were spread out evenly in sterile Petri dishes (8 cm) filled with filter paper. A number of germinated seeds in each dish was counted every day. The germination rate, expressed as an average time (days) needed for the single seed germination, was carried out. At the end of the test (21 days after sowing the seeds) the results were recorded as a germination percentage (%). 

### 3.5. Molecular Analysis

#### 3.5.1. Genotyping

DNA was extracted from young healthy leaves of 9–10 plants from wild-growing populations and 14 plants from ‘Lubelski’ landrace. The tissue was dried in active silica gel and ground into a fine powder in a bead mill (Mixer Mill MM 200, Retsch, Haan, Germany). The Genomic Mini AX Plant kit (A&A Biotechnology, Poland) was used for the extraction. The DNA quality was assessed in 2% agarose gel, and the quantity and purity were assessed using a spectrophotometer (NanoDrop ND-1000, Marshall, Hampton, VA, USA). Genotyping was performed using the DArTseq method in a commercial laboratory (Diversity Arrays Technology Laboratory, University of Canberra, Australia) which developed this technology. DArTseq™ is a combination of the DArT complexity reduction methods and sequencing on Illumina NGS platforms [50,51,52]. The genome complexity reduction was carried out with two restriction enzymes, namely MseI and PstI. The ligation of the corresponding adaptors was then performed and the resulting fragments were subsequently amplified [53].

#### 3.5.2. Data Analysis

The SNP markers were scored as binary data, indicating the presence (1) or absence (0) of a marker in the genomic representation of each sample, as described by Cruz et al. [50], and then the reproducibility (%), call rate (%) and polymorphism information content (PIC) were tested. The PIC was calculated as the average of the values obtained for all loci, in accordance with the following equation:

(1) $$PIC=1-\overset{n}{\underset{i=1}{\sum }}p_{i}^{2}$$

where *p_i_* is the relative frequency of the *i*th allele of the SNP loci. The genetic distance was measured using the unbiased Nei coefficient [51], according to the formula:

(2) $$uD=-\ln\left(\frac{\sum_{i=1}^{n}p_{ix}p_{iy}}{\sqrt{\left(\frac{2n\left(\sum_{i=1}^{n}p_{ix}^{2}-1\right)}{2n-1}\right)\times \left(\frac{2n\left(\sum_{i=1}^{n}p_{iy}^{2}-1\right)}{2n-1}\right)}}\right)$$

where *pix* and *piy* are the frequencies of the *i*th allele in populations *x* and *y* and *n* is the sample size. In order to determine the genetic structure, the agglomerative hierarchical clustering (AHC), according to Ward’s method, the principal coordinate analysis (PCoA) and the model-based Bayesian clustering algorithms were performed. To determine the number of clusters (K), the admixture and the correlated allele frequency model was used. Ten independent runs were set for each K value within the range of 1–10 with a burn-in period of 10,000 and 50,000 Markov chain Monte Carlo replications after the burn-in without prior information about the origin of the sample. The most probable value of K was determined using the method described by Evanno et al. [46]. The annual precipitation and the average annual temperature at the accessions’ collection sites were determined on the basis of the GPS coordinates using the WorldClim Version 2.0 database [54]. A correlation between the genetic distance and all of the other distance matrices was tested using the Mantel test (10^3^ permutations). The analyses were performed using Microsoft Excel 2016, XLSTAT Ecology (Addinsoft, Inc., Brooklyn, NY, USA), GenAlEx 6.501 [55], STRUCTURE v2.3.4 [56], CLUMPAK [57] and QGIS. The model-based Bayesian clustering was made as a part of the Computational Grant (G72-19) of the Interdisciplinary Centre for Mathematical and Computational Modelling, University of Warsaw (ICM UW).

### 3.6. Statistical Analysis

The data were subjected to a statistical analysis using the Statistica 12 software (Kraków, Poland). The mean values were compared using the one-way analysis of variance (ANOVA), by Tukey’s test, then expressed as mean with the standard deviation (±SD). The differences between the individual means were considered to be significant at *p* < 0.05.

## 4. Conclusions

The results indicate a high level of variability among the wild-growing common valerian populations, in respect to the developmental and chemical traits, but not strictly related to their geographical localization. In turn, the molecular analysis showed such an association, where five gene pools covering the regions of the population’s origin were observed, with the obvious distinctiveness of those located in the Bieszczady Mountains, as the most isolated. The gene flow was noticed within and between all examined regions, which indicates the affinity of the common valerian widespread in Poland. In the study, the wild-growing populations, recognized earlier as diploids, were compared to the ‘Lubelski’ landrace (tetraploid), a high-yielding model form, rich in sesquiterpenic acids. Among the populations, the one from the Mazowian Lowland (No. 18) and Bieszczady (No. 5), appeared to be closely related to the landrace, which was reflected in their highest mass of underground organs, followed by the highest content of sesquiterpenes. In the case of population No. 18, such a similarity was visibly associated with the huge admixture of the gene pool, typical for the ‘Lubelski’ landrace. Taking into account the results of the molecular analysis and the ploidy levels, it may be suspected that population No. 18 is a hybrid between the di- and tetraploid forms. Irrespective of the genetic relationship, the wild-growing populations were characterised by a high amount of essential oils in the underground organs, which may be promising from the point of view of practice. However, the sowing value of the seeds obtained from the examined populations, expressed as, the germinability and germination rate, was rather low. 

The present work confirms the thesis that the wild-growing populations of the common valerian may constitute a valuable source of genes for future cultivating programs. It also indicates the need of further screening of natural resources of the species, to find pure and/or hybrid polyploid accessions, usually characterized by a higher level of heterozygosity, in comparison with the diploids. This is especially important when considering the inbreeding tendencies within the wild-growing populations, followed by a degradation of their natural habitats associated with the recently observed climate changes, which altogether make the common valerian a threatened species.

## Figures and Tables

**Table 1:** Characteristics of the common valerian populations, in respect of the developmental and chemical traits of the underground organs.

	Developmental Traits		The Content of the Biologically Active Compounds	
	Rhizome Diameter (cm)	Mass of Underground Organs (Roots + Rhizomes) (g FW × plant^−1^)	Sesquiterpenic Acids (%)	Essential Oil (mL/kg)
Wild-growing populations				
1	3.4 ± 0.78 c	107.4 ± 36.9 d	0.007 ± 0.001 de	9.0 ± 1.2 a
2	4.5 ± 0.99 ab	164.8 ± 32.7 c	0.010 ± 0.001 c	9.8 ± 1.2 a
3	4.1 ± 1.56 b	166.3 ± 50.3 c	0.006 ± 0.001 e	6.7 ± 0.9 bc
4	5.5 ± 1.27 a	161.1 ± 45.8 c	0.006 ± 0.001 e	7.2 ± 1.0 b
5	6.0 ± 1.62 a	403.6 ± 119.2 a	0.031 ± 0.003 a	6.9 ± 1.0 bc
6	4.6 ± 0.99 ab	196.1 ± 31.3 bc	0.008 ± 0.001 d	7.8 ± 1.0 b
7	3.3 ± 0.36 c	131.1 ± 31.9 d	0.010 ± 0.001 c	8.0 ± 1.0 b
8	3.5 ± 0.80 c	186.6 ± 66.2 bc	0.004 ± 0.000 e	5.4 ± 0.3 c
9	5.1 ± 1.42 ab	220.7 ± 41.3 b	0.017 ± 0.002 b	5.4 ± 0.9 c
10	3.7 ± 0.74 c	252.5 ± 56.2 b	0.010 ± 0.001 c	10.4 ± 1.4 a
11	3.9 ± 0.74 b	160.8 ± 51.2 c	0.011 ± 0.001 c	7.7 ± 1.0 b
12	1.7 ± 0.59 d	112.8 ± 30.3 d	0.012 ± 0.001 c	6.0 ± 0.8 bc
13	3.9 ± 1.13 b	170.6 ± 61.8 c	0.005 ± 0.001 e	4.3 ± 0.6 cd
14	3.1 ± 0.61 c	247.7 ± 86.6 b	0.008 ± 0.001 d	8.1 ± 1.1 b
15	4.6 ± 0.92 ab	245.8 ± 76.5 b	0.009 ± 0.001 d	3.9 ± 0.5 d
16	5.5 ± 0.43 a	265.1 ± 55.9 b	0.009 ± 0.001 d	7.3 ± 1.0 b
17	5.1 ± 0.97 ab	187.8 ± 48.5 bc	0.008 ± 0.001 d	4.3 ± 0.4 cd
18	4.8 ± 1.10 ab	331.2 ± 88.5 ab	0.094 ± 0.009 a	5.1 ± 0.7 c
19	3.9 ± 0.60 b	190.8 ± 68.4 bc	0.010 ± 0.001 c	9.4 ± 1.2 a
mean	4.22	205.4	0.014	7.0
‘Lubelski’ landrace				
CV	0.24	0.35	1.35	0.27
	5.4 ± 1.24	375.0 ± 64.5	0.175 ± 0.018	6.3 ± 0.9

* Values in columns marked with different letters differ at *p* < 0.05; n = 10.

**Table 2:** Characteristics of the common valerian population’s aboveground organs.

No	Plant Height (cm)	Number of Flowering Shoots per Plant	Plant Lushness ^a^	Anthocyanin Coloring of the Shoots ^b^	Color of the Flower Buds ^c^	Color of the Petals ^d^	Teething of the Leaf Margin ^e^	Width of the Leaf Sections ^f^
Wild-growing populations								
1	135 ± 3 ab	15 ± 2 a	3	2, 3	3	2, 3	1, 2	1
2	110 ± 1 c	5 ± 2 d	1, 2	1, 2	2	2	2	1, 2
3	107 ± 7 c	7 ± 2 c	1, 2	1, 2	2	1, 2	1, 2, 3	1, 2
4	122 ± 5 b	10 ± 1 b	2, 3	1	2, 3	1, 2	1, 3	2
5	124 ± 6 b	12 ± 2 ab	2, 3	1	1, 2	1, 2	2, 3	1
6	124 ± 2 b	8 ± 0 c	1, 2, 3	1, 2	1, 2	1, 2	2, 3	1
7	134 ± 10 ab	11 ± 2 b	1, 3	1, 2, 3	1, 2	1, 2	1, 2	1
8	129 ± 6 b	14 ± 3 a	2, 3	2, 3	2	1, 2	1, 2	1
9	122 ± 6 b	9 ± 1 c	1, 2, 3	2, 3	2	1, 2	2	1
10	125 ± 9 b	9 ± 1 c	1, 2	2, 3	2	2	2, 3	1, 2
11	122 ± 9 b	13 ± 1 ab	1, 3	2	1, 2	1, 2	2, 3	1
12	120 ± 4 b	8 ± 2 c	1, 2, 3	1, 2, 3	1, 2	1	1, 2, 3	1, 2
13	134 ± 11 ab	11 ± 1 b	2, 3	1, 2, 3	1, 2	1	2, 3	1, 2
14	117 ± 2 c	11 ± 1 b	2, 3	1, 2	1, 2	1, 2	2	1
15	131 ± 3 ab	10 ± 0 b	2, 3	2	1, 2	1, 2	2	1, 2
16	133 ± 0 ab	5 ± 0 d	1	1, 2	2, 3	1, 2	1, 2	1, 2
17	122 ± 1 b	13 ± 1 ab	2, 3	1, 2, 3	1, 2	1, 2	2	1
18	143 ± 3 a	12 ± 1 ab	2	1, 2, 3	1, 2	1	1, 2	1
19	114 ± 6 c	11 ± 1 b	2	2, 3	1	1	1, 2	1
mean	124.63	10.21	-	-	-	-	-	-
CV	0.07	0.27	-	-	-	-	-	-
‘Lubelski’ landrace								
	128 ± 7	13 ± 1	2, 3	1, 2, 3	1, 2	1, 2	1, 2	1

* Values in columns marked with different letters differ at *p* < 0.05; n = 10; ^a^: 1—high; 2—medium; 3—low; ^b^: 1—absent; 2—medium intense; 3—intense; ^c^: 1—white; 2—pale pink; 3—pink; ^d^: 1—white; 2—pale pink; 3—pink; ^e^: 1—shallow; 2—medium deep; 3—deep; ^f^: 1—narrow; 2—medium wide; 3—wide.

**Table 3:** Seed parameters.

	1000 Seeds Mass (g)	Germinability (%)	Germination Rate (days)
Wild-growing populations			
1	0.2805 ± 0.0369 c	44.50 ± 3.23 c	6.00 c
2	0.4785 ± 0.0493 a	47.75 ± 1.10 c	7.39 b
3	0.4210 ± 0.0362 a	45.50 ± 3.34 c	7.73 b
4	0.4393 ± 0.0442 a	47.75 ± 1.96 c	8.10 b
5	0.1380 ± 0.0223 d	42.25 ± 2.71 c	6.11 c
6	0.3335 ± 0.0367 b	60.25 ± 2.59 a	13.00 a
7	0.3295 ± 0.0396 b	34.25 ± 3.51 cd	8.76 b
8	0.2765 ± 0.0335 c	30.25 ± 1.16 d	7.00 bc
9	0.3468 ± 0.0402 b	28.75 ± 1.09 d	7.00 bc
10	0.2890 ± 0.0218 c	52.00 ± 3.42 b	5.88 c
11	0.3375 ± 0.0260 b	48.00 ± 1.92 c	7.75 b
12	0.3233 ± 0.0291 b	56.50 ± 3.77 ab	6.45 c
13	0.2933 ± 0.0323 bc	53.00 ± 2.85 b	6.54 c
14	0.3538 ± 0.0312 b	25.25 ± 1.03 d	5.43 a
15	0.3270 ± 0.0353 b	58.25 ± 3.63 ab	4.64 a
16	0.3623 ± 0.0417 b	62.25 ± 2.55 a	6.39 c
17	0.3080 ± 0.0496 b	44.25 ± 3.08 c	8.41 b
18	0.3348 ± 0.0330 b	53.50 ± 2.89 b	11.14 a
19	0.3665 ± 0.0532 b	42.75 ± 1.24 c	6.82 bc
mean	0.3336	46.16	7.40
CV	0.21	0.22	0.26
‘Lubelski’ landrace			
	0.6463 ± 0.0582	81.25 ± 3.49	6.10

* Values in columns marked with different letters differ at *p* < 0.05.

**Table 4:** Geographical coordinates of the common valerian populations.

Population No	Region	Accession No.	Latitude	Longitude	Altitude
1	Noteć River Valley	401930	N 53 03 102	E 17 11 506	50
2	Bieszczady Mountains	401935	N 49 26 752	E 22 03 516	475
3	Bieszczady Mountains	403179	N 49 21 325	E 22 09 286	600
4	Bieszczady Mountains	403181	N 49 25 056	E 22 07 692	450
5	Bieszczady Mountains	403182	N 49 39 298	E 21 59 682	350
6	Bieszczady Mountains	403183	N 49 36 334	E 22 12 478	360
7	Bieszczady Mountains	403184	N 49 41 278	E 22 15 037	275
8	Nida Basin	403186	N 50 43 330	E 20 31 539	250
9	Nida Basin	403187	N 50 43 059	E 20 31 474	250
10	Nida Basin	401938	N 50 46 849	E 20 32 340	235
11	Nida Basin	401939	N 50 52 805	E 20 56 694	375
12	Kielce Upland	401941	N 50 93 333	E 20 56 673	290
13	Kielce Upland	401942	N 51 01 020	E 20 26 141	260
14	Kielce Upland	403190	N 51 01 025	E 20 28 164	260
15	Kielce Upland	401945	N 51 04 365	E 20 23 061	250
16	Kielce Upland	401946	N 51 14 630	E 20 22 354	210
17	Kielce Upland	403191	N 51 06 126	E 20 15 914	225
18	Mazovian Lowland	406738	N 52 07 282	E 21 05 541	80
19	Mazovian Lowland	403192	N 52 07 254	E 21 05 421	80
20	‘Lubelski’ landrace	404920	-	-	-

## Data Availability

All data is contained within the article.
